# A single sequence of intermittent hypoxia does not alter stretch reflex excitability in able‐bodied individuals

**DOI:** 10.1113/EP091531

**Published:** 2024-02-14

**Authors:** Andrew Q. Tan, Christopher Tuthill, Anthony N. Corsten, Stella Barth, Randy D. Trumbower

**Affiliations:** ^1^ Department of Integrative Physiology University of Colorado Boulder Colorado USA; ^2^ Department of Physical Medicine and Rehabilitation Harvard Medical School Boston Massachusetts USA; ^3^ Department of Physical Medicine and Rehabilitation INSPIRE Laboratory Spaulding Rehabilitation Hospital Boston Massachusetts USA

**Keywords:** acute intermittent hypoxia, spasticity, stretch reflex

## Abstract

Spasticity attributable to exaggerated stretch reflex pathways, particularly affecting the ankle plantar flexors, often impairs overground walking in persons with incomplete spinal cord injury. Compelling evidence from rodent models underscores how exposure to acute intermittent hypoxia (AIH) can provide a unique medium to induce spinal plasticity in key inhibitory pathways mediating stretch reflex excitability and potentially affect spasticity. In this study, we quantify the effects of a single exposure to AIH on the stretch reflex in able‐bodied individuals. We hypothesized that a single sequence of AIH will increase the stretch reflex excitability of the soleus muscle during ramp‐and‐hold angular perturbations applied to the ankle joint while participants perform passive and volitionally matched contractions. Our results revealed that a single AIH exposure did not significantly change the stretch reflex excitability during both passive and active matching conditions. Furthermore, we found that able‐bodied individuals increased their stretch reflex response from passive to active matching conditions after both sham and AIH exposures. Together, these findings suggest that a single AIH exposure might not engage inhibitory pathways sufficiently to alter stretch reflex responses in able‐bodied persons. However, the generalizability of our present findings requires further examination during repetitive exposures to AIH along with potential reflex modulation during functional movements, such as overground walking.

## Abstract (highlights)

- **What is the central question of this study?**
  Acute intermittent hypoxia (AIH) has been shown to enhance walking performance in persons with spinal cord injury but might also trigger changes affecting dysregulated stretch reflex pathways, leading to spasticity. We examined the effect of AIH on stretch reflex excitability in able‐bodied individuals.
- **What is the main finding and its importance?**
  We did not find significant changes in reflex responses after AIH nor changes in reflex scaling from passive to active conditions. This study provides a crucial insight that enhances our understanding of AIH engagement of key inhibitory pathways that affect reflex excitability.

## 1 INTRODUCTION

Spinal cord injury (SCI) often results in the devastating loss of limb function and independence. Many spinal cord injuries manifest as incomplete (iSCI), offering at least some sparing of neural pathways important for the recovery of voluntary limb movements. Contemporary therapies attempt to elicit functional plasticity within these residual pathways. However, restoration of limb use after iSCI remains elusive owing, in part, to the spontaneous onset of abnormal muscle co‐activity that undermines the beneficial effects of these treatments.

It is well documented that stretch reflexes are altered after SCI, often defined in the classical sense as spasticity (Lance, 1980). As a result, abnormal stretch reflexes are often a therapeutic target in SCI rehabilitation (Gracies et al., 1997). Reduction in exaggerated stretch reflexes coincides with improvement in functional skills, such as overground walking (Scivoletto et al., 2008; Thompson & Wolpaw, 2015). Thus, treatments that limit or reverse these maladaptive changes without hindering gains in limb function might translate into enduring health benefits and improved quality of life for those living with iSCI.

Breathing moderate episodes of low oxygen [i.e., acute intermittent hypoxia (AIH)] holds promise as a treatment to increase functional limb movements in people with iSCI (Tan et al., 2020; Vose et al., 2022). A single sequence of AIH (15 episodes, 1.5 min intervals at 10.0% O_2_) enhanced ankle torque generation by >80% and was correlated with a 40% increase in ankle plantar flexor muscle activity in persons who had sustained iSCI for >1 year (chronic iSCI) before study participation (Trumbower et al., 2012). Given that ankle plantarflexion is crucial for overground walking (Neptune et al., 2001), subsequent studies assessed the extent to which AIH might also correspond to improved walking ability in people with iSCI (Hayes et al., 2014; Navarrete‐Opazo et al., 2017; Tan et al., 2021). The first randomized clinical trial, by Hayes et al. (2014), showed that participants with iSCI who received a single sequence (one session) of AIH had better performance on the 10 m walk test (10MWT) by 18% in comparison to both sham treatment and baseline. Acute intermittent hypoxia applied before skill‐based walking training also improved walking ability in persons with iSCI more than either treatment alone.

The underlying neural mechanisms that contribute to the AIH‐induced increase in functional limb movements in people with iSCI remain unclear. However, there is considerable evidence from rodent iSCI models that a sequence of AIH elicits episodic serotonin release onto spinal motor nuclei that upregulate brain‐derived neurotrophic factor (BDNF) within phrenic motor nuclei (Baker‐Herman & Mitchell, 2002). The results of these changes correspond to enhanced breathing function. Similar upregulation of BDNF and its high‐affinity receptor, TrkB, occur in non‐respiratory motor nuclei and correspond to increased locomotor function in rats with cervical iSCI (Lovett‐Barr et al., 2012). Successful translation of AIH as a potential adjuvant to SCI rehabilitation ultimately requires a detailed understanding of the neural mechanisms giving rise to improved motor function in persons with iSCI. Although this evidence seems to suggest that AIH strengthens excitatory transmission of motor output (Dale‐Nagle et al., 2010; Vinit et al., 2009), repetitive AIH might also trigger a change in inhibitory transmission via BDNF‐dependent cotransporter activation (Boulenguez et al., 2010; Mabrouk et al., 2011). Given that AIH enhances BDNF expression of somatic motor nuclei in rats (Lovett‐Barr et al., 2012) and enhances locomotor ability in both rats and humans with iSCI (Boulenguez et al., 2010), it is plausible that AIH might promote similar benefits on inhibitory transmission in humans with chronic iSCI.

Balance in excitatory and inhibitory spinal transmission plays a vital role in walking after chronic iSCI. Excitatory inputs initiate walking, whereas inhibitory inputs coordinate muscle activity and often remain compromised after chronic iSCI. The BDNF‐dependent upregulation of key cotransporters is important for inhibitory spinal transmission after injury and might alleviate spasticity (Gao & Ziskind‐Conhaim, 1995; Kaila, 1994; Yamada et al., 2004). Agonist–antagonist muscle co‐activation profoundly compromises limb use in persons with chronic iSCI (Hayes et al., 2014; Trumbower et al., 2017), contributing to the dependence on assistive devices and limited community ambulation. Disruption in the balance between inhibition and excitation might also underlie aspects of limited motor control, such as altered interlimb coordination during overground walking (Sohn et al., 2018; Thibaudier et al., 2020) after SCI. However, AIH‐induced improvements in walking might result in BDNF‐dependent mechanisms that restore inhibitory circuitry to ameliorate hyperexcitable stretch reflex activity in people with chronic iSCI.

The purpose of this study was to quantify the effects of AIH on reflex excitability in a cohort of able‐bodied individuals. Considering classical studies that observed a shift in the Hoffman reflex recruitment curve following hypoxic exposure, we hypothesized that AIH would increase the stretch reflex activity of ankle plantar flexors in uninjured able‐bodied individuals. To test this hypothesis, we quantified the change in plantar flexor reflex activity across a functional range of background muscle activity before and after a single sequence of AIH. We also predicted that the magnitude of the stretch reflex response would be augmented with increasing background activity. The results of this study provide an important first step towards understanding how a plasticity promoter (AIH) might influence the modulation of dysregulated spinal reflex mechanisms in people with chronic iSCI.

## 2 MATERIALS AND METHODS

### 2.1 Ethical approval

We conducted a single‐blind, crossover, randomized study design to quantify the effects of a single sequence of AIH on stretch reflex excitability in able‐bodied individuals. A total of 16 individuals were enrolled in the study, and 14 of those completed the protocol. This trial was approved by the Mass General Brigham Human Research Committee's institutional review board (2017P001940) and was registered on ClinicalTrials.gov (NCT02323945) before participant recruitment. All participants provided written informed consent before engaging in study activities. All aspects of the present study adhered to the standards described in the *Declaration of Helsinki*.

### 2.2 Intervention

**FIGURE 1:**
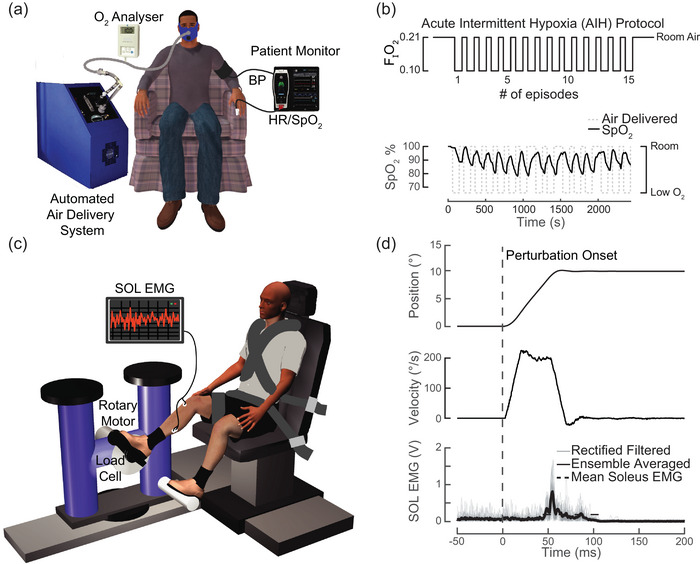
Experimental set‐up. (a) Low‐oxygen air was generated by pressure swing adsorption and delivered to participants via a non‐rebreathing mask. To ensure subject safety during interventions, SpO_2_, heart rate and blood pressure were monitored continually. (b) Participants received a single exposure of AIH (90 s hypoxic intervals of 10% F_I_O_2_ alternating with 60 s normoxic intervals of 21% F_I_O_2_) or normoxia. The SpO_2_ decreased in response to each AIH interval. (c) Participants were seated with their foot secured to a servomotor via a rigid footplate, with their knee fixed at 30° of flexion and ankle at 10° of ankle extension. Soleus EMG was recorded during maximal voluntary contractions and during stretch reflex perturbations. Perturbations were applied using a single degree‐of‐freedom brushless servomotor controlled in real time using MATLAB xPC. (d) Ramp‐and‐hold perturbations were applied to the ankle joint to induce a mechanical stretch reflex in the soleus muscle. Amplitude, velocity and acceleration were applied at 0.17 radians (10°), 3.5 radians/s (200°/s) and 200 radian/s^2^, respectively. Grey traces correspond to 20 overlaid rectified and filtered reflex responses. The perturbation‐evoked SOL stretch reflex is plotted as an ensemble average of the EMG responses. Abbreviations: AIH, acute intermittent hypoxia; F_I_O_2_, fractional inspired O_2_; SOL, soleus; SpO_2_, peripheral oxygen saturation.

Gas mixtures were delivered intermittently via two air generators (HYP123; Hypoxico) through computer‐controlled solenoid valves, as previously described (Tan et al., 2020). We monitored the air mixture delivery to participants to ensure delivery of the correct oxygen concentration (OM‐25RME; Maxtec; Figure 1a). Peripheral oxygen saturation and heart rate were measured continuously, and blood pressure was measured every fifth interval of hypoxia (RAD‐7, ROOT; Masimo) (Hayes et al., 2014; Tan et al., 2021; Trumbower et al., 2012). The participants were blinded to the intervention arm.

Participants were assigned randomly to receive a single day of AIH or sham intervention (SHAM). After a minimum of 1 week washout, participants then received the second intervention. This minimized the potential confounding effects of a single sequence of AIH (Trumbower et al., 2012). During the AIH intervention, participants received a single exposure of AIH (15, 1.5 min episodes at 0.10 ± 0.02 fractional inspired O_2_ with 1 min intervals; Figure 1b). The SHAM intervention included a single exposure to room air (15, 1.5 min episodes at 0.21 ± 0.02 fractional inspired O_2_ with 1 min intervals) using the same gas mixture delivery system. Stretch reflex responses were measured immediately before and after the AIH or SHAM intervention.

### 2.3 Muscle activity recordings

All participants underwent a pre‐ and postintervention maximal voluntary contraction (MVC) assessment for each sagittal plane ankle joint moment (i.e., ankle plantarflexion and dorsiflexion), as previously described (Finley et al., 2013; Trumbower et al., 2012). Participants sat in a modified long‐seated position, with their lower limb secured in 30° of knee flexion and 10° of ankle extension, such that the centre of rotation of their ankle aligned with the axis of rotation of the motor (Figure 1c). Straps secured the participant's waist, torso, thigh, ankle and distal foot to prevent voluntary changes in joint angle. We calculated the soleus muscle MVC by applying spline fit with a 50 ms window to the filtered EMG using a fourth‐order Bessel filter with an 80 Hz cut‐off. The MVC was defined as the 90th percentile of the filtered EMG signal. We computed the MVC from two, 3–6 s contractions for plantarflexion and dorsiflexion in separate trials.

We used a 16‐channel, 18‐bit data‐acquisition system (PCI‐DAS1602/16; National Instruments, USA) to record surface EMGs during MVC and reflex assessments at 2500 Hz. The EMG signals were amplified using a Bortec AMT‐8 system (Bortec Biomedical), which has a bandwidth of 10–1000 Hz, an input impedance of 10 GΩ and a common‐mode rejection ratio of 115 dB at 60 Hz. All analog signals were anti‐alias filtered using fifth‐order Bessel filters with a cut‐off frequency of 500 Hz. The EMG responses were recorded on the right soleus (SOL) using self‐adhesive disposable surface electrodes (Myotronics‐Noromed, USA), with a 2 cm interelectrode distance over the muscle bellies.

### 2.4 Stretch reflex assessment

We applied rotational, ramp‐and‐hold perturbations to the ankle joint against a fixed level of background EMG activation using a computer‐controlled brushless servomotor (Baldor BSM90N3 RF) to elicit soleus stretch reflexes. We controlled perturbation amplitude, velocity and acceleration parameters in real time using custom software with MATLAB xPC Target (v.2011b, RRID:SCR_001622). Participants were given a sequence of 20 perturbations, each with an amplitude of 0.17 rad (10°) and a velocity of 3.5 rad/s (200°/s), to elicit short‐latency reflex responses (Gottlieb & Agarwal, 1979) (Figure 1d). Owing to the acceleration dependence of short‐ and medium‐latency reflex responses (Finley et al., 2013), we used a constant perturbation acceleration of 140 rad/s^2^ for all trials. We recorded EMG responses for 5 s after the end of each perturbation.

Using real‐time visual feedback of their rectified soleus EMG, participants generated and matched a target soleus EMG amplitude that corresponded to three levels: 0 (passive), 10 and 20% of EMG generated during their MVC trials. Each target‐matching condition consisted of a block of 20 perturbations with the EMG target order randomized, for both pre‐ and postintervention. We programmed the ramp‐and‐hold perturbation to trigger only after the participant's EMG remained within 5% of the target value over a 250 ms time frame. Participants were given 10 s per trial to achieve the target EMG level. If they were unable to match the target level within 10 s, the trial was stopped and repeated. We instructed participants to not resist or react to the perturbation. The ankle was returned to the neutral position after 2 s, and there was a pause between perturbations with a duration selected randomly between 5 and 10 s.

### 2.5 Stretch reflex quantification

Surface SOL EMG was used to quantify the stretch reflex response of each participant to the applied ankle ramp‐and‐hold perturbations (Figure 1d). To account for any changes in background activity, we subtracted the passive background activity for the 50 ms period before each attempt at target matching. The soleus reflex responses were rectified and ensemble averaged to all 20 perturbations within each target activation block. To quantify the onset of the stretch reflex, we applied a moving SD filter with a 4 ms window to the rectified data. We defined the onset of the stretch reflex as the initial time following perturbation onset at which >50% of the next 2 ms of data exceeded 20% of the moving SD magnitude. The mean reflex amplitude was defined as the average EMG activity over a 120 ms window following the detected reflex onset.

### 2.6 Statistical analyses

All statistical analyses were performed using R Studio (v.2021.09.2, RRID:SCR_000432) at a two‐tailed significance of *P* = 0.05. Data in the text and figures are represented as mean values ± SD. To confirm that participants successfully met the EMG‐matching targets and that each condition was statistically distinct, a one‐way ANOVA was used to compare the preperturbation soleus EMG activation across each matching condition (passive, 10 and 20%). We compared differences of the soleus MVC before and after each intervention (SHAM/AIH) using Student's paired *t*‐tests.

We used a linear mixed‐effects model to quantify differences in the mean stretch reflex response while controlling for random effects (participant) and fixed effects (intervention and activation level). This approach allowed us to account for repeated measures within participants and to account for the three participants who did not complete the SHAM intervention arm. To examine the effect of AIH on the mean reflex amplitude, we constructed a linear mixed‐effects model to compare the ‘pre’ versus ‘post’ reflex response, with activation level (passive, 10 and 20%) as a fixed factor and participant as a random factor.

To assess the effect of intervention (AIH vs. SHAM), the mean EMG was normalized to the MVC magnitude to account for any differences between electrode placement or skin impedance between each session. A second linear mixed‐effects model was built, with normalized reflex responses and activation level as fixed effects and subject as the random effect. We used the Shapiro–Wilk test to assess whether the data were normally distributed and used Tukey's method for *post hoc* pairwise comparisons.

## 3 RESULTS

All 14 participants completed the AIH study rounds, and 11 participants completed SHAM study rounds; their demographics are listed in Table 1.

**TABLE 1:** Participant demographics.

Subject identity	Age (25.6 ± 4.3 years)	Sex
1	29	Female
2	26	Male
3	25	Female
4	34	Male
5	21	Female
6	19	Female
7	22	Female
8	22	Female
9	32	Female
10	30	Female
11	24	Male
12^a^	25	Male
13^a^	24	Male
14^a^	25	Male

^a^ Did not complete SHAM visit.

### 3.1 Maximal voluntary contractions

**FIGURE 2:**
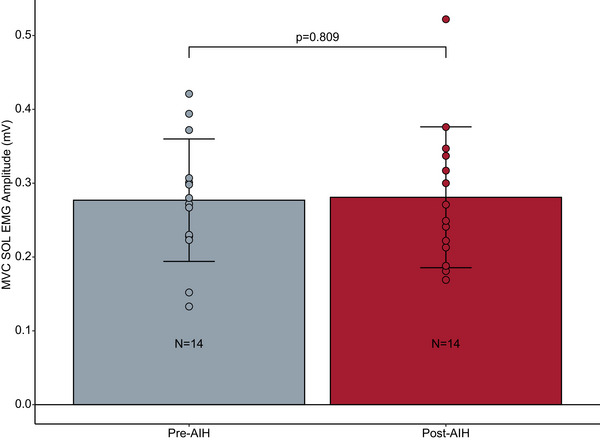
Effect of a single exposure to AIH on maximal voluntary SOL activation. The grey bar represents the mean SOL EMG activity before AIH exposure, and the red bar represents the mean SOL EMG activity after a single session of AIH. Error bars indicate the SD. Points represent individual participant data (*n* = 14). There was no significant difference between pre‐ and post‐AIH MVC amplitude (*P* = 0.809). Abbreviations: AIH, acute intermittent hypoxia; MVC, maximal voluntary contraction; SOL, soleus.

We did not find a significant difference in the SOL MVC activation before and after AIH (*P* = 0.809; Figure 2).

### 3.2 Reflex responses before AIH

All participants (*n* = 14) increased their mean SOL EMG activity for each target‐matching condition. Participants significantly increased their average SOL EMG amplitude from passive to the 10 and 20% matching conditions (*F* = 69.324, *P* < 0.0001), respectively. *Post hoc* contrast confirmed significant differences in SOL activation between all passive and active matching conditions (passive vs. 10%, *P* = 0.000201; passive vs. 20%, *P* < 0.0001).

**FIGURE 3:**
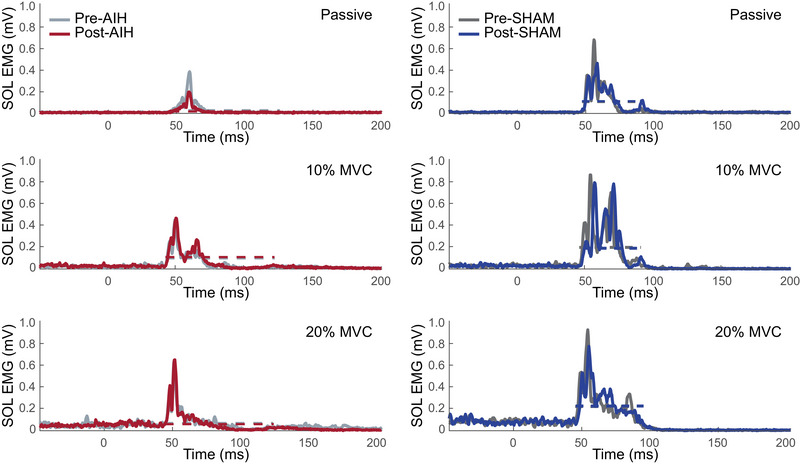
Electromyographic responses across treatments and background activation, illustrated by the stretch reflex response of the SOL EMG during AIH and SHAM conditions in a single participant. Each trace corresponds to the ensemble average of the filtered and rectified EMG response for all trials recorded for each of the conditions. Each row plots the reflex response during each activation condition where participants were asked to match the SOL EMG activation as a percentage of MVC. Grey lines correspond to the pre‐exposure response; coloured lines correspond to the postexposure response. Abbreviations: AIH, acute intermittent hypoxia; MVC, maximal voluntary contraction; SOL, soleus.

We also observed that the stretch reflex response amplitude increased with volitional activation. The normalized mean reflex response increased significantly (*F* = 58.12, *P* < 0.0001) from the passive to the active matching conditions (Figure 3). Specifically, the mean reflex response increased significantly from passive to 10% (*P* < 0.0001) and from passive to 20% (*P* < 0.0001). We found no significant difference in the mean reflex response during the 10 and 20% matching conditions (*P* = 0.902).

### 3.3 Reflex responses after AIH

**FIGURE 4:**
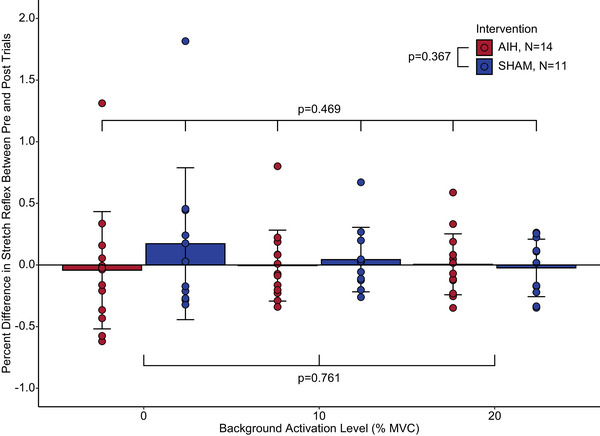
The effect of a single exposure to AIH and a single SHAM exposure to normoxia on the SOL stretch reflex response in able‐bodied participants. The mean reflex responses are normalized to the MVC for each condition and are shown as a percentage change from the pretreatment baseline. Blue bars indicate the mean group response after receiving the SHAM (normoxia) exposure (*N* = 11). Red bars indicate the mean group response after a single exposure to AIH (*n* = 14). Participants were asked to match SOL EMG activation conditions of 10 and 20% of MVC. Error bars indicate the SD, and points represent participant data. Abbreviations: AIH, acute intermittent hypoxia; MVC, maximal voluntary contraction; SOL, soleus.

A single exposure to AIH did not affect the stretch reflex response in able‐bodied individuals. In able‐bodied individuals, there was no main effect of treatment (AIH vs. SHAM; *F* = 0.825, *P* = 0.367) or activation level (*F* = 0.275, *P* = 0.761) on the mean, normalized reflex response (Figure 4). This indicated that a single session of AIH did not affect the reflex response. Furthermore, we found no significant interaction effect of treatment with activation level (*F* = 0.767, *P* = 0.469), indicating that AIH did not affect the scaling of the stretch reflex response during active conditions.

**FIGURE 5:**
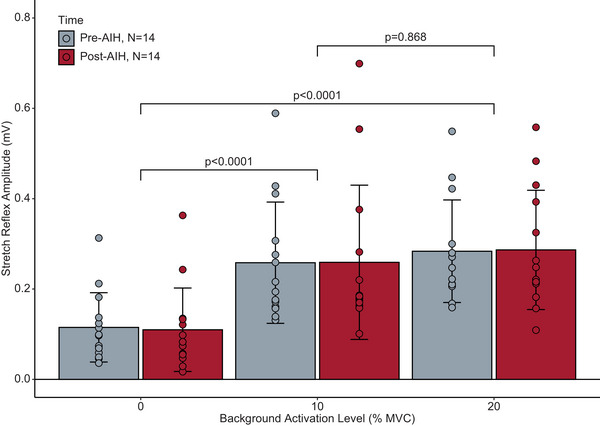
The effect of a single exposure to acute intermittent hypoxia on the SOL stretch reflex response in able‐bodied individuals. Grey bars indicate the mean group response before AIH exposure. Red bars indicate the mean group response after a single exposure to AIH. Error bars indicate the SD. The mean SOL reflex responses are plotted across each activation condition. Points represent participant data (*n* = 14). There was a significant difference (*P* < 0.0001) between passive and active conditions, but not between 10 and 20% MVC (*P* = 0.868). There was also no significant main effect of treatment on the mean reflex response (*P* = 0.973). Abbreviations: AIH, acute intermittent hypoxia; MVC, maximal voluntary contraction; SOL, soleus.

Likewise, a separate linear mixed‐effects model revealed that there was no significant main effect of treatment (Figure 5) on the mean reflex response (*F* = 0.001, *P* = 0.973). We found a significant main effect of activation level (*F* = 58.119, *P* < 0.0001). *Post hoc* comparisons indicated significant differences between the passive condition and both active conditions (10%, *P* < 0.0001; 20%, *P* < 0.0001) but not between the two active conditions (*P* = 0.868). Together, these results show that: (1) AIH did not affect the stretch reflex response in able‐bodied individuals; and (2) the increase of the reflex amplitude with greater activation was preserved across SHAM and AIH exposures. Acute intermittent hypoxia did not change the reflex modulation with volitional activation for either SHAM or AIH interventions.

## 4 DISCUSSION

In this study, we examined how a single exposure to AIH influenced the stretch reflex response of the SOL muscle in able‐bodied individuals. Additionally, we reported the effect of AIH on stretch reflexes during passive and active conditions using mechanical perturbations. We found that a single AIH exposure did not significantly change SOL stretch reflex response or MVC of participants. Furthermore, participants increased their SOL reflex activity with increasing SOL activation from the passive to active‐matching conditions after both SHAM and AIH. Here, we discuss several possibilities that might explain these findings and potential directions for future study.

### 4.1 Hypoxia‐induced changes in reflex excitability

The sensitivity of motoneurons to systemic exposure to hypoxia was first studied in intracellular recordings in spinalized preparations in the cat (Eccles et al., 1966; Gelfan & Tarlov, 1955; Kirstein, 1951). Both increases in the dorsal root potential during spinal cord anoxia (Gelfan & Tarlov, 1955) and spontaneous action potentials within afferent inputs have been reported (Kirstein, 1951). In a later study in cats ventilated with 5% oxygen, no significant changes were seen in the excitatory postsynaptic potentials recorded intracellularly from motoneurons (Eccles et al., 1966). These initial experiments suggest the insensitivity of the spinal monosynaptic reflex to hypoxia and the contribution of both pre‐ and postsynaptic elements.

In humans, earlier studies of the influence of hypoxia on the monosynaptic spinal reflex examined the SOL H reflex during a 20 min hypoxic exposure that produced an end‐tidal O_2_ concentration of 6%–7% (Willer et al., 1987). It was observed that although hypoxia had no effect on the slope of the H reflex recruitment curve, the threshold for both the H and M recruitment curves decreased, suggesting an increase in motoneuron excitability. Recently, these results were mirrored in a cohort of able‐bodied adults exposed to a single session of AIH, in whom a leftward shift in the H reflex recruitment curve was observed (Finn et al., 2022). Likewise, Schmeling et al. (1977) reported increases in the H and tendon jerk reflex at altitude in young, able‐bodied adults. In contrast, Willer et al. (1987) observed a hypoxia‐induced reduction in the H_max_/M_max_ ratio, which was presumed to be caused by an inhibitory supraspinal effect. We observed no effect of AIH on stretch reflex responses in able‐bodied individuals, which might be attributed, in part, to the differences in the dose of hypoxia and the differential excitation of stretch afferents. Given that the effect of AIH on the underlying neural correlates was not investigated, further study is required to examine how AIH‐induced effects on excitability are influenced by supraspinal and afferent modulation.

Another important consideration is that the reflex responses were obtained within a 90 min window after a single exposure to AIH. We chose this dose to align with previous studies, in which significant increases in corticospinal excitability were observed in able‐bodied individuals (Christiansen et al. 2018), in addition to increases in plantarflexion torque in persons with iSCI (Trumbower et al., 2012; Sandhu et al., 2019; Lynch et al., 2017) after only a single dose. In these studies, behavioural changes were observed ≤90 min after a single dose, indicating that our protocols were performed within this potential window of plasticity. Repetitive exposures to the same dose have also been documented to confer benefits on walking performance in persons with iSCI (Hayes et al., 2014; Tan et al., 2021), suggesting that the cumulative effects of repetitive treatments might affect the reflex response differentially.

Prior evidence also supports the role of BDNF in modulating motor responses by shaping GABAergic inhibitory neurotransmission that affects reflex responses linked with causing subsequent spastic motor behaviour after SCI. These studies in rodents (Tashiro et al., 2015) demonstrated how increased BDNF, via exogenous administration or activity‐based training, alleviates spasticity in rat SCI models by restoring inhibitory neurotransmission (Boulenguez et al., 2010; Tashiro et al., 2015). Notably, these studies also provide a direct link between increased BDNF influencing expression of the potassium chloride cotransporter (KCC2) that has been shown to play a key role in regulating spinal excitability by restoring appropriate inhibitory pathways after SCI (Boulenguez et al., 2010; Tashiro et al., 2015).

### 4.2 Activation‐dependent modulation of stretch‐sensitive reflexes

The amplitude of the stretch reflexes increased significantly from passive to active conditions. This observation is in accordance with previous findings of H reflex amplitude increasingly linearly during isometric contractions (Batista‐Ferreira et al., 2022; Burke et al., 1989; Matthews, 1986). The increase in H reflex excitability with activation is attributed to augmented descending drive, increasing the recruitment of motoneurons (Frigon et al., 2007; Pierrot‐Deseilligny, 1997). However, segmental mechanisms, such as reductions in presynaptic inhibition, can modify reflex modulation at contractions up to ∼50% MVC (Butler et al., 1993; Capaday & Stein, 1986) and might be affected by age (Klass et al., 2011; Obata et al., 2010). We observed significant increases in the stretch reflex response between passive and active conditions, but not between 10 and 20% active matching, although the preperturbation SOL EMG activation was significantly different. Furthermore, AIH did not affect the scaling of the stretch reflex with activation, suggesting that motor neuron pool excitability was not affected by either muscle activation condition after AIH. Prior studies indicate that longer‐latency components of the stretch reflex might involve adaptive transcortical mechanisms that scale with task complexity (Finley et al., 2012) and are affected by ageing (Kawashima et al., 2004; Klass et al., 2011). Although our assessment did not differentiate between short‐ and long‐latency components, the task challenge might not be sufficient to capture potential further differences in supraspinal contributions to the task.

### 4.3 Contraction dependence

Although our study characterizes responses only during isometric contractions in able‐bodied individuals, other groups have studied the influence of the type of contraction on reflex activity after SCI, including dynamic contractions of the ankle joint. For example, Kim et al. (2015) reported a lack of depression of the SOL H reflex during both passive and active lengthening of SOL in persons with SCI. This suggests that lengthening contractions further amplify already hyperexcitable Ia transmission. Both the changes in central activation (Kim et al., 2015) and differential recruitment of motor units during eccentric contractions (Duchateau & Enoka, 2008) might also influence the inhibition of Ia transmission after chronic SCI. Likewise, the level of active contraction might enhance the reflex responses (Burke et al., 1989). Although we observed a difference in the stretch reflex response from passive to 20% MVC, other groups have not reported differences between iSCI and able‐bodied participants in normalized H reflexes during passive conditions (Schindler‐Ivens & Shields, 2004) and active contractions at 75% MVC (Kim et al., 2015).

### 4.4 H reflex versus stretch reflex

Although the electrically elicited H reflex is used predominantly to study Ia excitation, the reflex induced by muscle stretch is thought to arise from the activation of less synchronous afferents, including group Ia and group II afferents (Jankowska & Edgley, 2010; McKeon & Burke, 1983; Morita et al., 1998), in addition to group Ib Golgi tendon organ afferents (Jankowska & McCrea, 1983; Nichols, 2018). Discrepancies between H reflex and stretch reflex modulation have been documented in both able‐bodied individuals (Morita et al., 1998; Sinkjaer et al., 1996) and persons with SCI (Thompson et al., 2019) during walking. A recent study comparing the SOL stretch reflex and H reflex during walking reported a weak correlation between the H reflex and the stretch‐induced reflex across the stepping cycle in persons with SCI, suggesting that the H reflex is not an adequate substitute for the stretch reflex (Thompson et al., 2019). Despite the differential sensitivity between the electrically elicited H reflex and the mechanically evoked stretch reflex, the modulation of each over the step cycle remained impaired. Thus, conclusions regarding their contribution to spastic gait must be interpreted carefully.

### 4.5 Study limitations

There are several important limitations associated with this study. First, our reflex perturbation parameters were chosen to maximize elicitation of a prominent short‐latency response. Changes in stretch perturbation parameters, such as acceleration and amplitude (Finley et al., 2013), have been demonstrated to alter the SOL stretch reflex response. Second, our reflex amplitude measurements do not differentiate between short‐ and long‐latency components. Partitioning the reflex response according to the onsets of short‐ and longer‐latency components might reveal further differences between segmental and supraspinal contributions to the reflex response (Kurtzer, 2014; Lee & Perreault, 2019). This distinction is especially crucial when evaluating reflex responses within dynamic environments that require task‐dependent muscle activation (Krutky et al., 2010; Shemmell et al., 2009). Indeed, prior studies have shown that more unstable loads at the ankle influence the pattern of both feedforward and feedback muscle activation (Finley et al., 2012). Importantly, our analyses focused only on isometric activation during passive and active matching tasks. Examining reflex behaviour during movement is essential to elucidate their contribution to perturbations during walking. As already mentioned, dynamic muscle contractions significantly influence the underlying excitability of the motor neuron pool and are an important topic for future study. Lastly, we did not characterize co‐contraction of the antagonist tibialis anterior muscle. Increases in antagonist muscle co‐contraction vary in a task‐specific manner (Lewis et al., 2010) but also affect the reflex response of the stretched muscles (Nielsen et al., 1994).

### 4.6 Clinical implications

The present findings indicate that a single session of AIH does not affect stretch reflex excitability or reflex modulation in able‐bodied individuals. This is in contrast to the findings of Christiansen et al. (2018), who demonstrated that a single session of AIH increases corticospinal excitability in able‐bodied participants. However, the magnitude of this effect might be time dependent (Finn et al., 2022). Our present observations suggest that a single session of AIH might not sufficiently activate BDNF‐dependent cascades that affect inhibitory spinal transmission to elicit observable change in the stretch reflex. It remains to be seen whether AIH affects reflex pathways in chronic human iSCI, thereby translating into clinically relevant reductions in spasticity in chronic iSCI. In addition to the loss of volitional control from descending pathways (Lin et al., 2012; Thomas et al., 1997), the presence of involuntary motor behaviour owing to spasticity (Adams & Hicks, 2005; Gorassini et al., 2004) can contribute to impairment of walking ability (Krawetz & Nance, 1996). Up to 78% of persons with chronic iSCI report symptoms of spasticity (Skold et al., 1999) and a velocity‐dependent increase in stretch reflex excitability (Skold et al., 1999), which contributes to a slower gait and angular joint velocity, reduced cadence (Krawetz & Nance, 1996), pain (Tibbett et al., 2020) and decreased range of motion (Skold et al., 1999). Although pharmacological treatments have been shown to reduce spasticity by targeting serotonin signalling, they also depress overall neural excitability and produce equivocal results in walking performance (Leech et al., 2014; Thompson & Hornby, 2013). It is promising that increases in ankle strength after AIH in persons with iSCI can be achieved (Trumbower et al., 2012) without an exacerbation of reflex hyperexcitability. Thus, AIH might be an effective treatment that can decrease spasticity while simultaneously enhancing motor recovery after iSCI.

## 5 CONCLUSIONS

In summary, able‐bodied persons demonstrated no change in the magnitude of the mechanically induced SOL stretch reflex after a single exposure to AIH. Additionally, we found that AIH did not affect the scaling of the reflex response with increasing volitional activation despite similar reflex amplitudes at 10 and 20% MVC. Further examination during dynamic movements and with the electrically induced Hoffman's reflex might be warranted in order to improve our understanding of the effect of AIH on spastic gait disorders. This study provides an important step towards an understanding of AIH engagement of key inhibitory pathways that affect reflex excitability and subsequently shape recovery of walking after iSCI (Aboubakr et al., 2001).

## AUTHOR CONTRIBUTIONS

Andrew Q. Tan and Anthony N. Corsten contributed to the design, implementation of the research, analyses of the results and the writing of the manuscript. Christopher Tuthill contributed to implementation of the research, analyses of the results and writing of the manuscript. Stella Barth contributed to implementation of the research and writing of the manuscript. All authors contributed to the interpretation of results. Randy D. Trumbower supervised and contributed to all aspects of the study. All authors approved the final version of the manuscript and agree to be accountable for all aspects of the work in ensuring that questions related to the accuracy or integrity of any part of the work are appropriately investigated and resolved. All persons designated as authors qualify for authorship, and all those who qualify for authorship are listed.

## CONFLICT OF INTEREST

The authors declare no competing financial interests.

## Data Availability

Data supporting this study are included within the article.
